# Gender and culture bias in letters of recommendation for computer science and data science masters programs

**DOI:** 10.1038/s41598-023-41564-w

**Published:** 2023-09-01

**Authors:** Yijun Zhao, Zhengxin Qi, John Grossi, Gary M. Weiss

**Affiliations:** Computer and Information Sciences Department, Fordham University, 113 W 60th St, New York, NY 10023 USA

**Keywords:** Human behaviour, Computational science, Computer science, Scientific data

## Abstract

Letters of Recommendation (LORs) are widely utilized for admission to both undergraduate and graduate programs, and are becoming even more important with the decreasing role that standardized tests play in the admissions process. However, LORs are highly subjective and thus can inject recommender bias into the process, leading to an inequitable evaluation of the candidates’ competitiveness and competence. Our study utilizes natural language processing methods and manually determined ratings to investigate gender and cultural differences and biases in LORs written for STEM Master’s program applicants. We generate features to measure important characteristics of the LORs and then compare these characteristics across groups based on recommender gender, applicant gender, and applicant country of origin. One set of features, which measure the underlying sentiment, tone, and emotions associated with each LOR, is automatically generated using IBM Watson’s Natural Language Understanding (NLU) service. The second set of features is measured manually by our research team and quantifies the relevance, specificity, and positivity of each LOR. We identify and discuss features that exhibit statistically significant differences across gender and culture study groups. Our analysis is based on approximately 4000 applications for the MS in Data Science and MS in Computer Science programs at Fordham University. To our knowledge, no similar study has been performed on these graduate programs.

## Introduction

Gender and cultural biases lead to inequity and many other societal problems. The impact of these biases on education is of great concern because education is one of the most effective ways of reducing these societal inequities or, put more colloquially, of “leveling the playing field”. For this reason, there has been increasing interest in ensuring that educational institutions do not perpetuate gender and cultural biases. Our study focuses on identifying bias in the university admissions process, with specific attention to Letters of Recommendation (LORs). These letters play an essential role in educational institutions’ holistic assessment of each candidate. However, LORs are highly subjective and hence can easily be influenced by the gender and cultural biases of the recommendation writer. The excerpted portions from two letters of recommendation from our study data, for the *same* female applicant, are quite informative:

Recommender 1: “$\dots$She came off as a diligent, well behaved and courteous student$\dots$ She is pleasant and has a personable nature.”

Recommender 2: “At a personal level, she is very bold and has never hesitated to voice her opinion without any fear. She has always taken a stand for herself and her friends in every situation and never steps back from her word.”

These statements provide vastly different impressions, which may be influenced by the first recommender being male and the second one being female. Research shows that the content and specific linguistic terms in LORs differ based on applicant gender and/or race and ethnicity, and that this impacts the impression of the applicant^1–8^. Another LOR in our study from an Asian country described the applicant as being “obedient,” a term unlikely to be found in a recommendation originating in the United States. Research also supports cultural differences in LOR^1,9^. These examples serve as one motivation for our work since they can exacerbate existing inequalities in higher education. The ability to identify such biases is a key step in addressing them.

This research focuses on gender and cultural biases in LORs for Computer Science and Data Science graduate programs. These disciplines are important because of rapid and continuing job growth, high pay, and the lack of trained workers; this study is particularly important because of the well-known gender disparities in these fields. This gender disparity is reflected in the United States Department of Education statistics^10^, which indicate that women earned only 33.4% of STEM MS degrees in 2021, and by our study that shows that only 37.7% of Fordham University MS Computer Science and Data Science applicants are female.

While there has been some research into bias in LORs, as described in the related work section, this study extends existing research and makes the following contributions:

- It covers bias in LORs of two STEM disciplines that have not been studied previously, thereby extending the research beyond medical education, which has been the main focus of prior research.
- More LORs (nearly 4000) are analyzed than in most prior studies.
- Cultural bias is studied along with gender bias, whereas most prior research focuses exclusively on gender bias.
- It uses the IBM Watson Natural Language Understanding (NLU) service to extract a dozen tones and emotions from the LOR text and analyses the differences based on gender and culture; prior work uses a smaller set of automatically generated features.
- The specificity, relevance, and positivity of each LOR is manually rated using well-defined guidelines and analyzed for differences in the gender and culture groupings. This is unique to our study.

The remainder of this paper is organized as follows. We first provide relevant background and related work. Next, we describe our data and preprocessing procedures. Then in the Methods section we introduce the features employed by this study and the procedures for extracting their values from the LORs. We then present the results and follow this with a discussion and a conclusion.

## Background and related work

This section describes work that is relevant to our study, including theoretical foundations, linguistic categories that have been used in the past to analyze bias, and prior LOR bias studies for graduate medical and science programs.

### Foundations of bias and relevant linguistic terminology

Gender bias in letters of recommendation can be explained by Eagly’s social role theory^11,12^, which states that beliefs about gender come from the social roles of men and women. People develop different beliefs about what men and women can do based on general observations of how they behave or are expected to behave. Society perceives men to be *agentic* (assertive, decisive) while women are perceived to be *communal* (benevolent, unselfish). Because achievement is deemed to be more closely associated with agency, this gender bias tends to favor men over women. Agency and communalism are well established in the psychological literature and provide a conceptual framework for understanding personality and behavior^13^. These terms are also relevant when discussing race, as racial inequality and the associated power differential lead to the same perceptions as are observed for gender^14^.

The biases just described are often reflected in linguistic differences. *Grindstone* words have to do with being hardworking or diligent (e.g., conscientious), while *standout* words have to do with being outstanding, excellent, or superb. Grindstone words, while seemingly positive, are often viewed as a “backhanded compliment”^15^ and are associated with weak recommendations^8^. Studies of bias in LORs often measure the prevalence of grindstone and standout words, and the underrepresented population that is presumably subject to bias is typically described using more grindstone and fewer standout words. Some studies also measure *doubt raisers*, which are negative phrases, hedges, or faint praise, such as “$\dots$she worked hard on the projects she accepted”.^8^ Numerous LOR bias studies^3,16^ use the Linguistic Inquiry and Word Count (LIWC)^17^ tool for automatically categorizing the linguistic content of text.

Cultural bias also impacts LORs. The schema theory of reading comprehension proposes that “the structures embodying background knowledge provide the ideational scaffolding for understanding” and “readers who share the cultural background of the writer come equipped with the appropriate schemata.”^18^ Another study confirms the role of cultural bias by stating that interpreting LORs is always difficult, “but especially if the cultural background of the referee and that of the reader are not the same”.^9^ That study compared LORs from the US and Asia and found differences in how the three main parts (introduction, body, and closing) were written^9^; for example, Asian recommenders were more likely to include a recommendation in the introduction. Another difference was that American recommenders were more likely than their Asian counterparts to provide indirect recommendations, such as “I hope that you will give favorable consideration to his application,” which are more likely to be misunderstood by non-native English speakers^19^.

### Studies of bias in medical LORs

Research on bias in letters of recommendation is heavily focused on graduate medical and professional programs, which are often viewed as biased toward White and Asian men. A study of 735 Duke University radiology residency applicants showed statistically significant differences in agentic and communal terms in LORs, with Whites and Asians showing higher agency than other racial groups, and women showing more agency than men^3^. This last observation may confound gender norms, but many of the agentic terms had to do with competence, and other research has shown that in such cases women may be viewed favorably^20^.

Two studies, however, support traditional gender biases. A study of 460 urology residency applicant LORs shows that letters written for male applicants contain significantly more references to personal drive, work, and power than those written for female applicants^2^, and a study of 332 surgical residency applicants shows that standout and agentic words most often applied to male applicants, while communal words, grindstone words, and doubt raisers are most often applied to female applicants^7^. Another study of 339 general surgery residents reinforced the racial differences identified in the Duke University study^3^ since it found that Black and Hispanic/Latinx applicants are more likely to be described by communal terms than agentic terms^5^.

### Studies of bias in STEM LORs

To our knowledge, there are no LOR bias studies for graduate Computer Science or Data Science programs, so we focus on the most similar population that has been studied, namely graduate (non-medical) science programs. A 2016 study of 1224 international geoscience postdoctoral fellowship LORs, which claimed at the time to be the largest study of bias in STEM LORs, classified the overall content of the letter as “Excellent”, “Good”, or “Doubtful”, and also tracked the length of the LORs^1^. Their analysis showed that female applicants were only half as likely to receive “Excellent” letters as their male counterparts, with no statistically significant interaction with recommender gender. Neither recommender gender nor applicant gender had a statistically significant impact on LOR length. The study showed, however, that the recommender’s region did make a significant impact on LOR length, with LORs written in America being shorter than those from other regions^1^.

A study of 886 chemistry and biochemistry faculty LORs showed that the LORs for male applicants included significantly more standout and ability words and fewer grindstone words than for the female applicants^6^, results that generally agree with the study of geoscience postdocs described earlier. A study of LORs for job applicants in the area of experimental particle physics, an area dominated by men, demonstrated a somewhat different pattern, in that LORs written by women were more favorable toward women than toward men^15^.

## Data and preprocessing

The data set for our study was constructed from 3,868 LORs extracted from six years of application data from Fordham University’s Master’s in Computer Science and Master’s in Data Science programs. Both Master’s programs are operated by the Computer and Information Sciences Department. For federal reporting purposes, each applicant is required to specify their gender using the binary “male” and “female” values. Using these binary values, 63% specify “male” and “37% specify “female,” which is consistent with the gender imbalance in these disciplines. Applicants also may optionally specify their gender identity using the following six options: “cisgender woman”, “cisgender man”, “transgender woman”, “transgender man”, “non-binary”, “prefer to self-identify”, and “prefer not to disclose”. Our study assigns the gender of the applicant based on the binary gender field because: the gender identity field is optional and hence sometimes missing, when it is specified it is set to the cisgender options in well over 99% of the cases, and some of the values like “non-binary” require separate analysis that is not feasible given the size of our data set. We recognize this is a limitation of our study as gender is analyzed only as binary and because transgender applicants may specify “gender” based on birth sex or gender identity (the application instructions imply it should be based on birth sex for federal reporting reasons but nonetheless refers to the field as “gender”). The gender identity distribution is roughly consistent with US Census Bureau survey data from 2021 that reports $0.6\%$ of adults identify as transgender^21^.

Our study includes the gender of the LOR writer for some of our analyses. Unlike an applicant’s gender, this information is not always available within the application. Thus, we implemented a multi-step approach to infer the missing gender information of recommenders. We first considered the recommender’s honorific (Dr., Mr., Mrs., Ms.) provided in the recommender information section and assigned the gender if it was gender-specific (e.g., Mr. or Ms.). This approach worked for some academic recommendations and most work-related recommendations. Alternatively, when the honorifics did not provide a clear indication (e.g., Dr.), we assigned the gender based on the predominant association of the LOR writer’s first name and cross-referenced the assignment with the recommender’s website, if available. In cases where both of these methods failed to provide a confident gender assignment, we resorted to analyzing the pronouns used on the relevant web pages of the recommenders. If none of the above steps enabled us to determine the LOR writer’s gender, we excluded those LORs from pertinent analyses. Using this procedure, we could not infer the recommender gender for 493 LORs, which accounts for approximately 13% of the total. As we made gender assignments only when we were confident in their accuracy, we believe the process has an overall high level of accuracy.

Our applicant and recommender gender data yield some interesting observations. First, only 29.5% of the LORs are written by women, which is lower than the percentage of female applicants (37.3%). This may be explained by the fact that many of the LORs are from academia, which has an even lower percentage of women than industry. Secondly, 35.1% of LORs for female applicants are written by women, while only 25.8% of LORs for male applicants are written by women, representing a 30.5% relative difference in the selection of female recommenders. This observation that applicants preferentially seek out recommenders of the same gender is an important result in itself, which suggests either a conscious or unconscious bias among applicants that they will receive the best recommendation from a recommender of the same gender. This preference for recommenders of the same gender was also noted in a study by Dutt et al.^1^, in which the authors reported $18.5\%$ ($9.4\%$) of female (male) postdoctoral geoscience fellowship applicant LORs being written by women, which represents a $32.6\%$ relative difference.

This study was approved by Fordham’s Institutional Review Board. All procedures were carried out following relevant guidelines and regulations. To comply with the Family Educational Rights and Privacy Act (FERPA), we anonymized student and recommender identities by automatically redacting their names and removing affiliations. Nonetheless, even with these safeguards, the LORs are still far too sensitive to share publicly; recommendation writers expect that the letters will remain confidential and no automated redaction system can ensure that the details described in the LOR will be insufficient to identify a recommender or applicant. Because this study is based on proprietary applications to Fordham University and there are no available public LOR data sets, researchers interested in this work can only confirm the findings indirectly, such as by applying the same methods to their application data. However, we consider direct reproducibility a lesser issue for this work because we do not make any claims about the superiority of our specific *methods* over other methods, and the results we have obtained at this stage merit sharing with a broader audience.

**Table 1:** Groups and variables to study LOR biases.

Study Groups	
- 2x2 groups by applicant and recommender genders	
- Applicant country groups (US, China, India)	
**Variables**	
Manual ratings	${{\textrm{NLP Tones}}^{1}}$
Positivity	t-Excited
Relevance	t-Frustrated
Specificity	t-Impolite
${{\textrm{NLP emotions}}^{1}}$	t-Polite
e-Anger	t-Sad
e-Disgust	t-Satisfied
e-Fear	t-Sympathetic
e-Joy	${{\textrm{NLP}}^{1}}$
e-Sadness	Sentiment

$^1$ Analysis performed using IBM NLU Tookit^22^. “e-” and “t-” indicate emotion and tone variables, respectively.

## Methods

In this study We investigated differences in LORs across different gender and culture groups, which may indicate bias. The variables employed in this study include three manually rated features, as well as emotions, tones, and sentiment scores automatically extracted using NLP (Natural Language Processing) models provided by the IBM Watson NLU service. Previous studies have demonstrated the effectiveness of NLP software in capturing the nuances of emotion and tones expressed in various contexts. For instance, it has been used to analyze online reviews of orthopedic surgeons^23^, identify gender bias in LORs for general surgery residency candidates^24^, and evaluate teachers’ written feedback to students^25^. While it can be argued that most LORs are typically written in a positive manner, our research and a prior study^24^ consistently reveal statistically significant differences in linguistic characteristics, including “joy” and “sadness,” among LORs for different gender groups. Recognizing the significance of these subjective expressions and their potential impact on readers, we employed the IBM NLU package and tone analyzer to analyze all emotion and tone categories. Table 1 lists the study groups and the 16 variables utilized in our study.

This study was approved by Fordham’s Institutional Review Board (IRB@fordham.edu), and informed consent was waived. All procedures were carried out following relevant guidelines and regulations.

### Manually rated features

Our data set includes three manually assigned scores that collectively summarize the overall quality and strength of the recommendation. These features, which measure three different dimensions of each LOR, are *relevance*, *specificity*, and *positivity*. They are each assessed independently and assigned specific values based on a set of carefully constructed guidelines, which include sample LORs that outline the expected ratings for each feature, along with explanations for the assigned values. Table 2 provides a summary of the possible ratings for each feature, accompanied by brief descriptions. To minimize variability among raters, they participated in practice sessions where they rated a set of sample LORs and discussed any discrepancies to ensure consistent interpretations of the guidelines and calibration of ratings. A team of ten research assistants generated the final ratings over a span of two months. As an example, the following LOR text would have a recommended relevance of “Minimal” and specificity of “Excellent” because it is not very relevant to our two STEM degree programs but contains many specific details:

“$\dots$ REDACTED goes above and beyond and would often take on a leadership role when it came to responding to the staff email alias. she is very proactive and dedicates time to reviewing departmental budgets and address any concerns that may lead to deficits. $\dots$”

While all 16 variables in Table 1 are interesting, our experience suggests that the three manually rated features hold significant importance in determining the overall strength of a recommendation.

**Table 2:** Definition of manually rated LOR features.

Value	Description
**Positivity**	
Weak (0)	Trying to put positive spin
Positive (1)	Several positives but below average
Strong (2)	More positive than average
Very Strong (3)	Unusually positive; exceptional
**Relevance**	
Minimal (0)	Little relevant info for making decision
Good (1)	Some relevant info for making decision
Excellent (2)	Extremely helpful for making decision
** Specificity**	
Poor (0)	Form letter with almost no specifics
Average (1)	only a few specifics; knows casually
Good (2)	Several specific statements
Excellent (3)	Many specifics; knows applicant well

### Generating features using NLP software

There has been a revolution in the application of NLP with the advent of toolkits and pre-trained models provided by leading companies such as Google^26^, OpenAI^27^, Microsoft^28^, and IBM^22^. In this study, we subscribed to the Natural Language Understanding (NLU) service provided by IBM Watson, which uses deep learning to extract meaning and metadata from unstructured text, including categories, classification, entities, keywords, sentiment, emotion, relations, and syntax. We leveraged this service to infer the tones and emotions in each LOR. The tones are described by seven sub-scores (polite, satisfied, sympathetic, excited, frustrated, sad, and impolite), each of which ranges from 0 to 1. The definition of each tone is provided in the tone analytics section of the IBM Watson NLU documentation^22^ as follows:

- *Excited*—showing personal enthusiasm and interest
- *Frustrated*—feeling annoyed and irritable
- *Impolite*—being disrespectful and rude
- *Polite*—displaying rational, goal-oriented behavior
- *Sad*—an unpleasant passive emotion
- *Satisfied*—an affective response to perceived service quality
- *Sympathetic*–an affective mode of understanding that involves emotional resonance

Emotions are described using five categories (anger, disgust, fear, joy, and sadness) with values that range from 0 to 1. Note that according to NLU’s scheme, sadness is an emotion while sad is a tone.

The last variable used in our study is the sentiment score, which quantifies the LOR writer’s overall attitude toward the applicant. It is a continuous value that ranges from most negative ($-1$) to neutral (0) to most positive ($+1$). A high score of 0.9444 was generated for a LOR with text “$\dots$ *based on my experience with numerous high-tech experts, I can fully say that his intelligent thinking, teamwork attitude and analytical skills are exceptional asset to my team* $\dots$,” while a low score of $-0.5395$ was generated for an LOR with text “$\dots$ *frankly, I have little memory of him or his work since nothing was submitted in computer readable format in those days* $\dots$ *Chris’ grade of A- put him in the top 20% of that class (18 students, I believe)* $\dots$” This example illustrates that although most recommenders write favorably about the applicant, the NLP model is capable of detecting nuances of overall sentiment, similarly for the emotion and tone variables employed in this study.

The Emotion and Sentiment features were extracted using the default NLU model (version 2022-04-07), while the seven tone scores were obtained using the English tone analytics model (tone-classifications-en-v1). Since the tone model only analyzes the first 2000 characters of text, we split more than half of the LORs into smaller components so the full text could be analyzed. To this end, NLTK’s sentence tokenizer was employed to ensure that all splits occurred at sentence boundaries. The final score for each letter is the average of all component scores weighted by the length of each component.

## Results

In this section we analyze the LOR features across our study groups. We first compare the LORs by applicant gender and identify statistically significant features among the variables outlined in Table 1. We are also interested in learning if any observed applicant biases are present among the male and female recommenders. We next conduct a parallel study where we swap the roles of recommender and applicant gender and compare the LORs by recommender gender and examine these differences while controlling the applicant gender. Lastly, we present the findings across three culture groups (i.e., US, China, and India).

### Comparing LORs by applicant gender while controlling for recommender gender

Table 3 summarizes the differences between LORs written for female and male applicants, with further analysis on the recommender’s gender. The results only include features for which the differences by applicant gender are statistically significant with a 95% confidence.

Without considering recommender gender, the results for “All Recommenders” show six features with statistically significant differences between the male and female applicant groups. Female applicants receive longer letters (4%) with higher positivity (11%) and specificity (7%), and with less anger ($-2$%). However, the tones of these letters are less polite ($-5$%) and sadder (4%).

We are interested in learning if these differences originated from the male or female recommenders. To determine this, we control the study samples by recommender gender. The results in the corresponding “Female Recommenders” and “Male Recommenders” blocks show that both female and male recommenders provide more specific and positive letters for female applicants. However, this difference is more pronounced among female recommenders, who show a 16% higher positivity and 12% higher specificity score for female applicants over male applicants, while the respective differences for male recommenders are only 10% and 6%. Since positivity and specificity are among the key features influencing the overall recommendation, our findings suggest that female applicants receive stronger letters than their male counterparts, especially from female recommenders. Although not directly comparable, our results agree with other studies that show women often receive more favorable LORs^15^.

Another notable observation is that female applicants tend to receive 7% longer letters than male applicants from female recommenders—a phenomenon that is not observed among male recommenders. This length difference is particularly notable because prior research has demonstrated that LOR length is positively related to its favorability^29^ and psychological experiments have shown that longer LORs are written when the object of the letter is viewed more favorably^30^. Interestingly, a study of LORs written by school counselors for college applicants^31^ shows that female recommenders wrote longer letters than their male counterparts for *male* applicants, while both groups wrote similar length LORs for female applicants, thus showing a different gender bias compared to our study. Our findings also indicate that the LORs for female applicants are associated with a less polite tone, observed across all three control groups in Table 3. Prior studies have not focused on politeness in writing styles and we feel this pattern is worthy of further research.

**Table 3:** Comparison of LOR features by applicant gender while controlling for recommender gender.

Category	Applicant		%Diff	*p*-value
	Female	Male		
	All recommenders			
Applications	1433	2435	–	–
Length (chars)	2293	2198	4%	0.0038
Positivity	1.4417	1.2854	11%	$<10^{-4}$
Specificity	1.6490	1.5347	7%	0.0001
e-Anger	0.0603	0.0616	$-2$%	0.0037
t-Polite	0.3084	0.3256	$-5$%	$<10^{-4}$
t-Sad	0.1486	0.1424	4%	0.0058
	Female recommenders			
Applications	447	542	–	–
Length (chars)	2402	2241	7%	0.0155
Positivity	1.5257	1.2970	16%	$<10^{-4}$
Specificity	1.7494	1.5590	12%	0.0004
e-Anger	0.0591	0.0615	$-4$%	0.0054
t-Polite	0.3061	0.3242	$-6$%	0.0242
t-Sad	0.1420	0.1317	8%	0.0068
	Male Recommenders			
Applications	825	1561	–	–
Positivity	1.4024	1.2735	10%	0.0002
Specificity	1.6097	1.5234	6%	0.0242
t-Polite	0.3068	0.3243	$-6$%	0.0013

**Table 4:** Comparison of LOR features by recommender gender while controlling for applicant gender.

Category	Recommender		%Diff	*p*-value
	Female	Male		
	All Applicants			
Applications	991	2398	–	–
Length (chars)	2312	2190	5%	0.0017
Positivity	1.3986	1.3161	6%	0.0068
Specificity	1.6418	1.5505	6%	0.0051
e-Anger	0.0604	0.0614	$-2$%	0.0459
e-Fear	0.1000	0.1021	$-2$%	0.0042
e-Joy	0.4506	0.4448	1%	0.0043
e-Sadness	0.2404	0.2433	$-1$%	0.0200
t-Excited	0.1398	0.1299	7%	0.0006
t-Sad	0.1364	0.1492	$-1$%	$<10^{-4}$
Sentiment	0.7591	0.7375	3%	$<10^{-4}$
	Female Applicants			
Applications	447	825	–	–
Length (chars)	2402	2222	8%	0.0027
Positivity	1.5257	1.4024	8%	0.0095
Specificity	1.7494	1.6097	8%	0.0051
e-Anger	0.0591	0.0610	$-3$%	0.0184
t-Excited	0.1411	0.1286	9%	0.0070
t-Sad	0.1420	0.1515	$-7$%	0.0120
	Male applicants			
Applications	542	1561	–	–
e-Fear	0.1002	0.1023	$-2$%	0.0339
e-Joy	0.4501	0.4440	1%	0.0228
t-Excited	0.1388	0.1307	6%	0.0325
t-Sad	0.1317	0.1477	$-12$%	$<10^{-4}$
Sentiment	0.7656	0.7372	4%	$<10^{-4}$

**Table 5:** LOR feature comparison between applicants from the United States (US), China (CH), and India (IN).

Features	Group Mean			*p*-value				
	US	CH	IN	ANOVA	Bartlett’s	Tukey HSD test		
						US vs CH	US vs IN	CH vs IN
	All applicants							
Length (chars)	2163	2456	2715	$<10^{-4}$	$<10^{-4}$	0.0010	0.0010	0.0075
Positivity	1.3105	1.5144	1.5817	$<10^{-4}$	$<10^{-4}$	0.0010	0.0010	0.5979
Specificity	1.5288	1.8298	1.7908	$<10^{-4}$	$<10^{-4}$	0.0010	0.0010	0.8487
e-Disgust	0.0394	0.0395	0.0342	$<10^{-4}$	$<10^{-4}$	0.9000	0.0010	0.0010
e-Fear	0.1027	0.1000	0.0936	$<10^{-4}$	$<10^{-4}$	0.0023	0.0010	0.0010
e-Joy	0.4402	0.4511	0.4840	$<10^{-4}$	$<10^{-4}$	0.0010	0.0010	0.0010
e-Sadness	0.2445	0.2426	0.2254	$<10^{-4}$	$<10^{-4}$	0.3439	0.0010	0.0010
t-Excited	0.1306	0.1317	0.1548	0.0004	0.0183	0.9000	0.0010	0.0012
t-Impolite	0.0170	0.0116	0.0155	$<10^{-4}$	$<10^{-4}$	0.0010	0.2235	0.0010
t-Polite	0.3183	0.3244	0.2914	0.0105	$<10^{-4}$	0.4504	0.0252	0.0073
Sentiment	0.7377	0.7417	0.7935	$<10^{-4}$	$<10^{-4}$	0.7607	0.0010	0.0010
	Female applicants							
Length (chars)	2241	2519	2419	0.0001	$<10^{-4}$	0.0001	0.3248	0.7322
Specificity	1.6332	1.8339	1.7031	0.0023	0.0003	0.0015	0.7996	0.5068
e-Disgust	0.0395	0.0393	0.0356	0.0374	0.0059	0.9854	0.0283	0.0545
e-Fear	0.1019	0.1006	0.0913	0.0002	0.0002	0.6058	0.0001	0.0019
e-Joy	0.4446	0.4489	0.4881	$<10^{-4}$	0.0003	0.4615	$<10^{-4}$	$<10^{-4}$
e-Sadness	0.2458	0.2404	0.2276	$<10^{-4}$	$<10^{-4}$	0.0033	$<10^{-4}$	0.0019
t-Excited	0.1330	0.1263	0.1605	0.0052	0.0361	0.3939	0.0144	0.0034
t-Sympathetic	0.0784	0.0969	0.0931	$<10^{-4}$	0.0039	$<10^{-4}$	0.0056	0.7318
Sentiment	0.7429	0.7310	0.7953	0.0061	$<10^{-4}$	0.4460	0.0147	0.0040
	Male applicants							
Length (chars)	2119	2423	2928	$<10^{-4}$	$<10^{-4}$	$<10^{-4}$	$<10^{-4}$	$<10^{-4}$
Positivity	1.2420	1.4738	1.6517	$<10^{-4}$	$<10^{-4}$	$<10^{-4}$	$<10^{-4}$	0.1238
Specificity	1.4688	1.8272	1.8539	$<10^{-4}$	$<10^{-4}$	$<10^{-4}$	0.0001	0.9598
e-Disgust	0.0393	0.0396	0.0332	$<10^{-4}$	$<10^{-4}$	0.8527	$<10^{-4}$	$<10^{-4}$
e-Fear	0.1032	0.0997	0.0952	$<10^{-4}$	$<10^{-4}$	0.0023	0.0008	0.1234
e-Joy	0.4379	0.4524	0.4810	$<10^{-4}$	0.0001	$<10^{-4}$	$<10^{-4}$	$<10^{-4}$
e-Sadness	0.2458	0.2404	0.2276	$<10^{-4}$	$<10^{-4}$	0.0033	$<10^{-4}$	0.0019
t-Impolite	0.0168	0.0118	0.0154	$<10^{-4}$	$<10^{-4}$	$<10^{-4}$	0.4013	0.0084
t-Polite	0.3244	0.3336	0.2923	0.0117	$<10^{-4}$	0.3027	0.0435	0.0094
t-Sad	0.1455	0.1334	0.1526	0.0005	0.0124	0.0009	0.5732	0.0277
Sentiment	0.7352	0.7474	0.7922	0.0007	$<10^{-4}$	0.2290	0.0009	0.0194

Statistically different features (Column 1) across applicant groups from three countries with a 95% confidence interval for the statistical tests. The *p*-values from the ANOVA and Barlett’s tests indicate if the three group means are statistically different. We infer the distinctive features in each group from the *p*-values of the Tukey HSD test (last three columns)

### Comparing LORs by recommender gender while controlling for applicant gender

This section investigates the differences between the LORs written by female and male recommenders. The results, presented in Table 4, include only those features for which the differences by recommender gender are statistically significant with a 95% confidence.

Without considering applicant gender, the results for “All Applicants” show that ten features exhibit statistically significant differences between the female and male recommenders. Compared to the letters from male recommenders, LORs from female recommenders are longer (5%), more positive (6%), more specific (6%), and the emotional content has less anger ($-2\%$), less fear ($-2\%$), more joy ($1\%$), and less sadness (-1%). Female recommenders also tend to write with a less sad ($-1\%$), more excited ($7\%$) tone and the overall sentiment score is 3% higher than their male counterparts.

When we limit the applicants to “Female Applicants,” we observe that five out of the ten features from the “All Applicants” block remained statistically significant, i.e., length ($8\%$), positivity ($8\%$), specificity ($8\%$), anger emotion ($-3\%$), together with excited ($9\%$) and sad ($-7\%$) tones. Furthermore, the differences in these features are more pronounced than those in the aggregated group. On the other hand, if we limit the applicant pool to “Male Applicants,” then there are only marginal differences in fear ($-2\%$) and joy ($1\%$) emotions, but more substantial differences in excited ($6\%$) and sad ($-12\%$) tones, and overall sentiment ($4\%$).

A closer examination of the two sets of variables in the female and male applicant blocks reveals that all recommenders used a more excited and less sad tone for female applicants. However, the two recommender groups show unique differences in specificity, positivity, and LOR length. In particular, only female recommenders provided higher scores to female applicants, and this discrepancy is observed across all three categories. This finding that female recommenders write stronger letters for female applicants than their male counterparts is consistent with the results presented in the previous section. Lastly, female recommenders showed more positive sentiment (4%) than male recommenders for the male applicants; however, this pattern is not observed for the female applicant group.

### Comparing LORs across culture groups

This section analyzes the differences in LOR characteristics across applicants from the United States (US), China (CH), and India (IN), which collectively cover most of our applicants. No other countries contribute a significant number of applicants.

We first identify variables whose group means are statistically different across the country-specific groups. To this end, we applied a one-way Analysis of Variance (ANOVA) test^32^ to each variable, which is an extension of the Student t-test for more than two study groups. Specifically, ANOVA compares the means among the groups and determines whether any of those means are statistically significantly different from each other. Formally, for our application, it tests the null hypothesis:

$$\begin{aligned} H_0: \mu ^i_{US} = \mu ^i_{CH} = \mu ^i_{IN} \end{aligned}$$

where $\mu ^i_{US}$, $\mu ^i_{CH}$, and $\mu ^i_{IN}$ denote the average value of variable $x_i$ over applicants in the US, China, and India subgroups, respectively. We also applied Bartlett’s test^33^ to ensure the homogeneity of variances assumption in the ANOVA analysis. Since a statistically significant result from an ANOVA analysis only indicates at least one group differs from the others, we next performed the Tukey HSD (“Honestly Significant Difference”) *post-hoc* test^34^ to identify where the significance lies in our culture groups.

**Figure 1:**
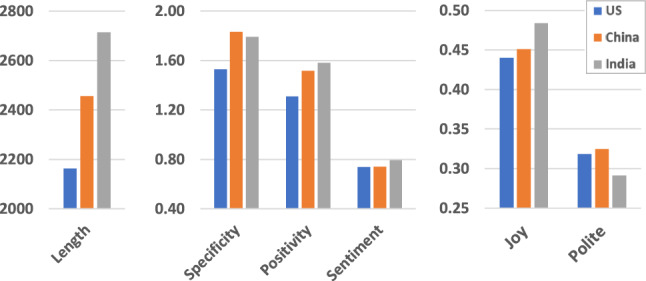
Characteristic features across country groups.

Table 5 presents the statistically significant features (Column 1) thresholded at a 95% confidence interval for ANOVA, Barlett’s, and Tukey HSD tests. The group means and *p*-values for each variable are displayed in columns 2–4 and 5–9, respectively. The last three columns present the pairwise *p*-values generated by the Tukey HSD test, from which we can infer the country group with a distinctive mean. For example, the pairwise *p*-values for specificity for “All Applicants” are 0.001, 0.001, and 0.8487 for the US vs. CH, US vs. IN, and CH vs. IN, respectively. Consequently, this feature is *significant* for US vs. CH and US vs. IN, but *not* statistically significant for CH vs. IN, suggesting that specificity is a distinctive feature for the US group. Figure 1 shows the inter-country comparison for a selected set of features for the all applicants group with three scales for clarity.

An analysis of the results for the “All Applicants” block in Table 5 yields the following key observations:

- The LORs for the US group are distinguished by low specificity and positivity.
- The Chinese group stands out for its low impolite score for the underlying tone.
- The India group is marked by more positive sentiment, less sad and disgust emotions, and a more excited tone.
- Three variables (length, joy, fear) are statistically different for all country groups.
- The US group has the shortest LORs (2163), followed by China (2456), and India (2715). Not only are all differences statistically significant, but they are substantial: US vs. CH (12.7%), US vs. IN (22.6%), and CH vs. IN (10.0%).

Our findings for LOR length are consistent with another study of a graduate STEM population, which showed that postdoctoral geoscience fellowship LORs originating from US recommenders were statistically significantly shorter than LORs from all other regions^1^. Interestingly, the pattern we found for LOR length is replicated for positivity, with India having the highest values, followed by China, and then followed by the US with the lowest LOR length and positivity values.

There is a general lack of prior cross-cultural results for the other LOR characteristics, but the low impoliteness score for China conforms to the more general observation of Chinese linguistic politeness, which is a result of Chinese tradition and the teachings of Confucianism that emphasizes propriety and humility^35^. Overall, the US group exhibits the highest “impolite” score with the India group a close second. One might expect a reverse pattern for politeness, but this is not observed as the US and China groups both have relatively high scores while India has a significantly lower politeness score. This discrepancy can be explained by the NLU definition of “polite,” listed in the Methods section as “displaying rational, goal-oriented behavior”; in this case “polite” and “impolite” are not antonyms.

We next delve deeper into the variations within each gender group (“Female Applicants” and “Male Applicants”) across three countries. Our results indicate that most observations from the previous analysis hold for both gender groups, although there are some exceptions. Additionally, in certain instances, these observations are more pronounced for one gender group. For example, the US group has the lowest specificity for both gender groups, but the difference between the US group and the China and India groups is more pronounced for the male applicants than the female applicants. This pattern continues for positivity, with statistically significant results observed only for male applicants. Furthermore, The low impolite score previously found for the China group is only statistically significant for the male applicants. The high positive sentiment previously observed for the India group holds for both applicant genders and the short LORs found for the US group also holds for both genders.

By comparing the gender-based differences across countries, we observe that the impact of gender is not always consistent. One notable difference is in the length of LOR, which we believe is one of the most important LOR features. Specifically, in the US and China groups, male applicants tend to receive slightly shorter LORs compared to their female counterparts. In contrast, for the India group, male applicants tend to receive significantly *longer* LORs, with an average of 2928 characters compared to 2419 characters for female applicants. A similar pattern is observed for specificity, where the male applicants for the US and China groups have lower LOR positivity values than those of their female counterparts. Conversely, in the India group, the male applicants receive *higher* specificity scores (1.8539 vs. 1.7031). Thus, for both features, the US and China groups have the more positive values assigned to the female applicants, while for the India group the more positive values are assigned to the male applicants. These findings highlight the varying impact of gender across different countries, which is consistent with the results from the Women’s Workplace Equity Index^36^. This index measures gender inequities on equality under the law in seven categories. Under the “finding a job” category, which is most relevant to this study, the US and China have similar scores, 57.1 and 53.3, respectively, while India has a much lower score of 37.5^36^. These gender differences across countries is worthy of additional study.

## Discussion

Our analyses in the Results section contend that overall (i.e., not considering the gender of the recommender), female applicants to our MS in Computer Science and MS in Data Science programs received stronger letters of recommendation than their male counterparts due to higher positivity ($11\%$) and specificity ($7\%$) scores and longer length ($4\%$). This finding disagrees with some prior studies that showed women tend to receive weaker recommendations. However, the vast majority of these prior studies assess the LORs primarily on the presence or absence of agentic, communal, standout, and grindstone words^4,6,8,15,37^. Our study focuses on different aspects using content-based criteria (i.e., length, relevance, specificity, and positivity) and linguistic components (i.e., tone and emotion). They distinguish our work from other word statistics-based studies but also make many results incomparable. For instance, we discovered unique gender and culture differences in the manually rated features, which provides a new perspective that is arguably more relevant to the recommendation quality and has not been explored in prior LOR bias studies.

Our observed results challenge the commonly held stereotype that women have lesser aptitude in math and science, particularly in non-health related fields such as computer science. Interestingly, a recent study found that “women are outperforming men in both physical and life science undergraduate courses at the same institution, while simultaneously continuing to be perceived as less-able students^38^. Of particular relevance is a study that showed that female students earned higher grades than their male counterparts in their MS in Data Science coursework at Fordham^39^. While the possible superiority of female applicants might suggest that the observed LOR differences may not solely be attributed to gender bias, our findings strongly suggest otherwise. Indeed, our results showed that the differences for LOR length, positivity, and specificity, which we believe have a great influence on the strength of the LOR, are *magnified* by the gender of the recommender. Specifically, for female applicants the values for those LOR features are much higher when the recommender is female rather than male—and the LOR length difference is not statistically significant when the recommender is male. Thus, it is highly unlikely that gender does not play a role in the observed variations. This suggests a disparity in the way male and female applicants are perceived and evaluated.

Our LOR data analysis showed that female applicants are $30.5\%$ more likely to seek out a female recommender than a male applicant. This preference for female recommenders among female applicants can be seen as a manifestation of gender empowerment. It indicates that female applicants may perceive female recommenders as role models or advocates who can better understand and support their experiences, challenges, and potential^40–42^. This can foster a sense of solidarity among women and contribute to building supportive networks. Regardless of the underlying reason, the preferential selection of female recommenders by female applicants is beneficial given the results in our study, which indicate that this decision is likely to result in a stronger overall recommendation. It is important to note that the social impact may vary depending on the context, cultural factors, and other intersecting identities, such as race, ethnicity, and socioeconomic status. Further research and analysis would be needed to fully understand the implications of these findings and their broader societal effects.

Cultural values and beliefs shape the way people express themselves, and these differences can impact how applicants are evaluated and perceived. The differences in specificity, tone, and sentiment indicate variations in communication styles across cultures. The low specificity and positivity in the US group may reflect a more general and less detailed approach to recommendation letters. The more positive sentiment and excited tone in the Indian group may reflect a cultural emphasis on enthusiasm and optimism. The variations in emotional expressions, such as sadness, disgust, joy, and fear, reflect the cultural differences in emotional norms and the importance of emotional expression. The higher positive sentiment and lower negative emotions in the Indian group, for example, may indicate a cultural tendency to emphasize positive emotions and downplay negative ones. The shorter LORs in the US group may suggest a preference for brevity and efficiency, while the longer LORs in the Indian group may reflect a more elaborate and detailed communication style. The gender differences observed in the India group, which tend to benefit the male applicants, which are not observed for the US and China groups, may reflect differing gender biases. Recognizing and understanding these cultural differences is generally important for effective cross-cultural communication and, in particular, is necessary for an accurate assessment of the LORs. However, cultural differences are extremely complex and can vary within countries and regions. Additional research and analysis are warranted to gain a more comprehensive understanding of the impacts of these cultural differences on recommendation letters and the evaluation process.

## Conclusion

This study analyzed the LORs for graduate STEM programs in Computer Science and Data Science and identified LOR characteristics that exhibit statistically significant differences based on applicant gender, recommender gender, and culture. Our study indicates that female applicants receive stronger letters than their male counterparts, and that this difference is much more extreme when the recommenders are female. The differences based on recommender gender indicate the presence of gender bias. Since our data indicate that applicants preferentially seek out recommenders of the same gender, the above finding further magnifies the impact of this gender bias. We further examined cultural differences, and the key findings are that the US group receives LORs that are on average shorter, less positive, and less specific than applicants from China or India. The China group stands out for its low impolite score for the underlying tone, while the India group is marked by more positive sentiment, less sad and disgust emotions, and a more excited tone.

As mentioned in the introduction section, our study makes several contributions to research on bias in letters of recommendation. Our study focuses on bias in MS Computer Science and MS Data Science LORs, which have not been studied in the existing literature, and the number of LORs analyzed is larger than for most other studies. Our study also investigates both gender and cultural biases, and is the first one that we are aware of that uses IBM Watson NLU service to analyze a dozen tones and emotions. Finally, our study explores the interaction of gender and culture with LOR specificity, positivity, and relevance, which was possible only after an extensive and time-consuming manual labeling process.

One limitation of this study is that it relies primarily on an empirical approach, where we report the statistically significant differences observed across a relatively large number of features. While certain features such as LOR relevance, specificity, and positivity were carefully chosen, others were selected based on the choice of NLP software. Although we attempted to justify and explain our findings based on existing psychological, social, and linguistic theories^11–14^, these theories only covered a subset of the features included in our study. Many of the differences in emotion and tone within the LORs remained unexplained by existing theories, indicating the need for further research in this area. Nevertheless, identifying and quantifying these gender and cultural differences is important, as they serve as a foundation for future investigation and research. Another limitation of this study is its reliance on a binary construct of gender, as discussed in the Data and Preprocessing section. While this choice has some influence on the study, its overall impact is minimal as the majority of applicants identify their gender based on their assigned sex at birth. In our case, the data collected limited our ability to explore additional gender categories. It would be interesting to extend future studies to incorporate a broader range of gender identities, especially since all of the studies referenced in this article only consider gender as a binary concept.

There are multiple avenues to extend the work presented in this paper. One avenue we are currently pursuing involves utilizing machine learning to develop a predictive model to automatically assess the specificity, positivity, and relevance of LORs. This approach would allow future research on bias to bypass the time-consuming manual labeling process employed in this study while facilitating the automatic evaluation of LOR quality. Although the primary focus of this research was to investigate gender and cultural differences and biases, our long-term objective is to contribute to bias reduction. To this end, we plan to develop an automated tool that can flag areas of potential bias for both letter writers and readers. This tool would enable stakeholders to either address the bias or, at the very least, be sufficiently aware of it to discount its influence. This objective holds significance, as our preliminary findings indicate compelling positive correlations between certain study variables and admission decisions, underscoring the meaningful impact of these biases on the admissions process. Another avenue we intend to pursue is the application of our methodology to various undergraduate and graduate programs to facilitate comparisons between programs. The findings can provide informative insights into the variations of biases across different educational contexts. Currently, making such comparisons is challenging, as existing LOR bias studies primarily focus on a single population and employ different methodologies. Finally, we found the cultural differences observed in the LORs interesting. However, due to the limitations imposed by the size and demographics of our program, we could only analyze three groups (US, China, and India). Exploring additional cultures and countries may yield different findings, enhancing our understanding of how cultural factors influence the content and characteristics of LORs.

## Data Availability

The data used in the current study is not publicly available due to its proprietary nature and compliance requirements with the Family Educational Rights and Privacy Act (FERPA). Researchers interested in accessing the data, please contact Dr. Yijun Zhao or Dr. Gary Weiss at Fordham University to discuss the possibility of gaining access through protocols such as a data usage agreement.
